# Irbesartan ameliorates inflammation via transendothelial leukocyte migration due to VCAM-1/NOX-1 signaling in cisplatin-induced cardiotoxicity

**DOI:** 10.22038/IJBMS.2023.70997.15422

**Published:** 2023

**Authors:** Dilek Ulusoy Karatopuk, Songül Özkula, Esra Aydoğdu, Halil İbrahim Büyükbayram, Adem Milletsever, Fatih Aksoy

**Affiliations:** 1 Department of Histology and Embryology, Faculty of Medicine, Süleyman Demirel University, Isparta, Turkey; 2 Department of Pharmacology, Faculty of Medicine, Süleyman Demirel University, Isparta, Turkey; 3 Department of Pharmaceutical Research and Development, Health Science Institute, Süleyman Demirel University, Isparta, Turkey; 4 Department of Biochemistry, Faculty of Medicine, Suleyman Demirel University, Isparta, Turkey; 5 Department of Pathology, Faculty of Veterinary Medicine, Mehmet Akif Ersoy University, Burdur, Turkey; 6 Department of Cardiology and Department of Pharmacology, Faculty of Medicine, Süleyman Demirel University, Isparta, Turkey

**Keywords:** Cardiotoxicity, Cisplatin, Irbesartan, VCAM-1, VEGF

## Abstract

**Objective(s):**

Cisplatin (CP) is frequently used in various types of cancers. The cardiotoxic effects of this agent limit its usage. Our study seeks to investigate the protective effects of Irbesartan (IRB) on CP-induced cardiotoxicity.

**Materials and Methods:**

The following four groups comprised thirty-two rats: control, CP, CP+IRB, and IRB. On the fourth day of the experiment, 5 mg/kg of CP was given to CP and CP+IRB groups intraperitoneally, and for seven days, water or IRB 50 mg/kg (orally) was administered. Vascular endothelial growth factor (VEGF), caspase-3 (Cas-3), vascular cell adhesion molecule-1 (VCAM-1), NADPH oxidase-1 (NOX-1), creatine kinase MB (CK-MB), and lactate dehydrogenase (LDH) were measured.

**Results:**

The levels of VCAM-1, NOX-1, VEGF, Cas-3, and LDH were increased in the CP group. The treatment with IRB decreased VCAM-1, NOX-1, VEGF, Cas-3, and LDH levels significantly (*P<*0.05). Histopathological examination revealed normal heart architecture in Control and IRB groups. While marked hyperemia and myocardial cell degeneration were noticed in the CP group, significant amelioration was observed in the CP+IRB group. Aortas in the CP group showed endothelial damage and desquamation. IRB treatment markedly ameliorated histopathological findings in the CP+IRB group. Cardiac and aortic damage caused by CP was attenuated by IRB treatment owing to the anti-inflammatory and antiapoptotic effects of IRB.

**Conclusion:**

IRB may help reduce the severity of CP-induced cardiac injury by limiting leukocyte migration and reducing inflammation and apoptosis.

## Introduction

Cisplatin (CP) or cisplatinum is a chemotherapeutic agent and is widely used to treat solid cancers such as testicular, cervical, bladder, ovarian cancer, and lymphomas (1, 2). The best known of the anticancer action mechanisms of CP is that it causes DNA damage by interacting with purine bases and induces apoptosis (1, 2). However, the side effects of CP, which is so widely used, limit its use (1). Among the side effects of CP are gastrointestinal toxicity, cardiotoxicity, ototoxicity, nephrotoxicity, myelotoxicity, and hepatotoxicity (2). Acute and cumulative cardiotoxicity is one of the significant side effects that limit the use of CP (3). Accumulating evidence indicates that vascular toxicity is one of the most critical late consequences of CP-based chemotherapy. Endoplasmic reticulum stress, inflammation, and apoptosis mechanisms are blamed for the pathogenesis of cardiovascular toxicity caused by CP (4, 5).

Additionally, it has been shown that the incidence of metabolic syndrome (hypertension, dyslipidemia, insulin resistance, and obesity) has increased in survivors following the cumulative dose of CP chemotherapy (6, 7). However, it has been revealed that higher inflammatory status constitutes an essential underlying factor of metabolic syndrome (8). CP has been shown to increase inflammatory status by increasing vascular cell adhesion molecule-1 (VCAM-1) expression and tumor necrosis factor-a (TNF-a) levels in the kidneys (9).

Several mechanisms have been involved in the pathophysiology of CP-induced toxicity, such as oxidative stress, apoptosis, and inflammation following CP treatment. Kim *et al. *(10) reported that activation of the MAPK pathway increases inflammation and intercellular adhesion molecule expression, VCAM-1, and E selectin. VCAM-1, which is a cell adhesion molecule, is expressed in endothelial cells and is known as endothelial cell surface glycoprotein. Although proinflammatory cytokines activate VCAM-1 expression, it is also released from other cells such as macrophages and myoblasts in chronic disease states (11). Thus, VCAM-1 has an essential role in the extravasation of leukocytes (11, 12) and contributes to developing diseases associated with inflammation (12). Therefore, many investigations recommend that the MAPK pathway can be used as a possible goal to investigate therapeutic interventions to reduce CP-induced toxicity.

Another mechanism explaining the underlying cause of CP-induced cardiotoxicity is an increase in reactive oxygen species and a direct toxic effect on cardiac myocytes (9, 13-15). The NADPH oxidase (NOX) enzymes are involved in pathological and physiological conditions and catalyze the formation of superoxide (16, 17). The NOX family mainly covers reactive oxygen species (ROS) production in the cardiovascular system (17). NOX-1, a member of the NOX family, is one of the most expressed NOX in vascular smooth muscle cells (16-18). ROS, which is formed due to the reaction in which NOX-1 plays a role, has essential contributions to physiological conditions such as angiogenesis, cell signaling, cell growth, and pathological conditions such as atherosclerosis, neurological disorders, and inflammation (16). A study (19) reported that NOX-1 expression is associated with CP-related ototoxicity.

Irbesartan (IRB), an angiotensin receptor blocker, treats hypertension and diabetic nephropathy (20). It is stated that angiotensin receptor blockers protect cardiac myocytes by alleviating inflammation (21). Furthermore, a study (22) showed that IRB was protective against diabetic kidney disease by suppressing inflammation. However, the protective mechanisms of IRB effect on the cardiovascular system are not entirely known. Therefore, the present experimental study was performed to assess its cardiovascular protective potential in CP-induced cardiovascular toxicity and further research on whether NOX-1/VCAM-1 signaling mediates this cardiovascular protection.

## Materials and Methods 

***Animals and ethical approval***

This study’s experiments followed the animal research guidelines of the National Institutes of Health, and the Committee on Animal Research of Süleyman Demirel University, Isparta approved the procedures (Approval No: 01.10.2020; 07; 09). Thirty-two female Wistar Albino rats with a weight range of 250 to 350 g were put in room-controlled humidity (60%±5%) and temperature (21 ^°^C-22 ^°^C) conditions and a 12:12 hr dark/light cycle was kept for them. A standard commercial chow diet (Korkuteli Yem, Antalya, TURKEY) was fed to all rats. 

***Study design and animals***

A total of 32 Wistar albino female rats with a weight range of 250 to 350 g were kept at %60±5 humidity, 22 ^°^C and a 12-hr dark/light cycle. All groups were fed *ad libitum*. The local animal experiments ethics committee of Isparta Suleyman Demirel University approved our experiment (ethics No: 01.10.2020/07-09). The rats were divided randomly into four groups: control, CP, CP+IRB, and IRB.

For the control group (n = 8), on the fourth day of the experiment, 1 ml of saline was given intraperitoneally, and for seven days, 1 ml of water was given by gavage.

For the CP group (n = 8), 7.5 mg/kg CP was administered intraperitoneally on the fourth day of the experiment, and for seven days, 1 ml of water was given by gavage (23, 24).

For the CP+IRB group (n = 8), 7.5 mg/kg CP was administered intraperitoneally on the fourth day of the experiment, and for seven days, 50 mg/kg IRB was orally administered by gavage (25).

For the IRB group (n = 8), on the fourth day of the experiment, 1 ml of saline was given intraperitoneally, and for seven days, 50 mg/kg IRB was orally administered by gavage.

Twenty-four hours after the last drug administration, 10 mg/kg Xylazine and 80 mg/kg Ketamine were administered to every rat. Blood was taken from vena cava inferior after sacrification. Cardiac and aortic tissue were harvested for histopathological, biochemical, and immunohistochemical analyses at the experiment completion.

***Biochemical analyses***

Centrifugated gel-containing tubes were used to contain rats’ blood. The temperature of the storage place of samples before analyses was -80 ^°^C. A spectrophotometric method using Beckman Coulter AU 5800 automated analyzer (Beckman Coulter, USA) was used to determine lactate dehydrogenase (LDH) and Serum Creatine kinase MB (CK-MB) levels.

***Genetic analyses***

*Reverse transcription-polymerase chain reaction (RT-qPCR)*

Following the manufacturer’s instructions, TRIzol^TM^ Reagent Monarch Total RNA isolation kit (New England BioLabs) was used to extract Total RNA from rat tissues. A MySPEC microvolume spectrophotometer (VWR) was used to determine RNA purity and concentration. The iScript cDNA Synthesis kit using oligo dT primers (Bio-Rad Laboratories, Hercules, CA, USA) was used to reverse transcribe 1 μg of RNA. The incubation duration and temperature for the reaction mix were 5 min, 20 min, and 1 min at 25 ^°^C, 46 ^°^C, and 95 ^°^C, respectively. Following the manufacturer’s instructions, iTaq Universal SYBR Green Supermix (Bio-Rad Laboratories, Hercules, CA, USA) was used to perform real-time PCR amplification. Also, a CFX96 instrument (Bio-Rad Laboratories, Hercules, CA, USA) found a fluorescence signal. In order to amplify VCAM-1 (Forward 5’-CCTGAACTCCTTGCACTCTAC-3’, Reverse 5’-CACCAGACTGTA CGATCCTTTC-3’), NOX1 (Forward 5’-TGAAGGATCCCATCAGAGAAAC-3’, Reverse 5’-CCCAACCAAGAAACCAGAAAC-3’), specific primers were designed. For each PCR, samples of cDNA were analyzed in triplicates. Normalization was conducted using the GAPDH expression. The conditions of PCR were as follows: after denaturation at 95 ^°^C for 10 min, 40 cycles were performed for 10 sec at 95 ^°^C, and for 30 sec at 60 ^°^C. The template was 100 ng cDNA, and 25 µl was the ultimate total reaction volume. The comparative ΔΔCt method was used to perform gene expression relative quantification. To ascertain the specificity of amplification, a melting curve was used to analyze the PCR products. The fold change in the graph was selected as the presentation method for all results.

***Histopathological and immunohistochemical analyses***

An automatic tissue processor (Leica ASP300S, Wetzlar, Germany) was used for the pathological examination procedure after the cardiac and aortic tissues were embedded in paraffin wax after fixing in %10 buffered formalin. A coverslip-mounted hematoxylin-eosin (HE) was used to remove the sections from the paraffin blocks. Then, they were stained and investigated via a microscope. Two sections series of all blocks of the cardiac and aortic tissues were drawn on poly-L-lysine coated slides and were stained immunohistochemically for cas-3 (Anticas-3 Antibody E-8:7272(Santa Cruz, Texas, USA) and VEGF (AntiVEGF Antibody JH121:57496) (Santa-Cruz, Texas, USA) expression by streptavidin-biotin technique. Streptavidin-alkaline phosphatase conjugate and biotinylated secondary antibody were used to perform immunohistochemistry after 60 min incubation with the primary antibodies. A specialized pathologist from another university performed analyses blindly. The score grading system ranging from (3) to (0) was used to evaluate cardiac and aortic tissues’ immunohistochemical semiquantitative status as follows: (3)=diffuse strong staining, (2)=diffuse weak staining, (1)=focal weak staining, and (0)=negative. The assessment was made in ten random areas using The Database Manual Cell Sens Life Science Imaging Software System (Olympus Co., Tokyo, Japan).

***Statistical analyses***

The SPSS 22.0 program pack (SPSS Inc., Chicago, IL, USA) was used for data analyses. Serum CK-MB, LDH levels, and genetic and immunohistochemistry scores were compared with one-way ANOVA. *P*<0.05 was considered significant.

## Results

***Biochemical results***

*Measurement of blood parameters*

**Figure 1:**
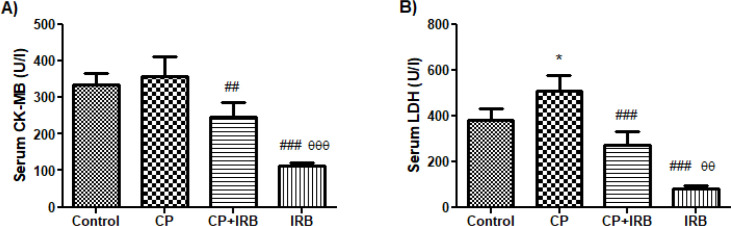
Levels of analyses of biochemical markers in the blood

CK-MB levels were decreased in CP+IRB and IRB groups significantly compared with the CP group (*P*<0.01 and *P*<0.001; respectively). CK-MB levels were reduced in the IRB group compared to the CP+IRB group (*P*<0.001). CK-MB levels were increased in the CP group compared with the control group, but it is not statistically significant (*P*>0.05; Figure 1). LDH levels were significantly higher in the CP group than in the control group (*P*<0.05). LDH levels were significantly decreased in CP+IRB and IRB groups compared with the CP group (*P*<0.001, for all). LDH levels were reduced in the IRB group considerably compared with the CP+IRB group (*P*<0.01; Figure 1). 

***Relative mRNA expression analysis by qRTPCR***

The relative mRNA level of VCAM-1 in CP increased significantly more than in the control group (*P*<0.001). The relative mRNA level of VCAM-1 in CP+IRB and IRB decreased significantly compared to the CP group (*P*<0.05, *P*<0.001; respectively). The relative mRNA level of VCAM-1 in IRB decreased significantly compared to the CP+IRB group (*P*<0.001).

**Figure 2:**
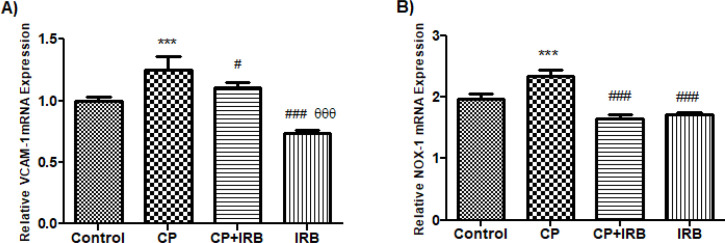
Levels of analyses of the qRT-PCR assays

The relative mRNA level of NOX-1 in CP increased significantly compared to the control group (*P*<0.001). The relative mRNA level of NOX-1 in CP+IRB and IRB decreased significantly compared to the CP group (*P*<0.001; for all) (Figure 2).

***Histopathological and immunohistochemical results***

*Histopathological results*

**Figure 3:**
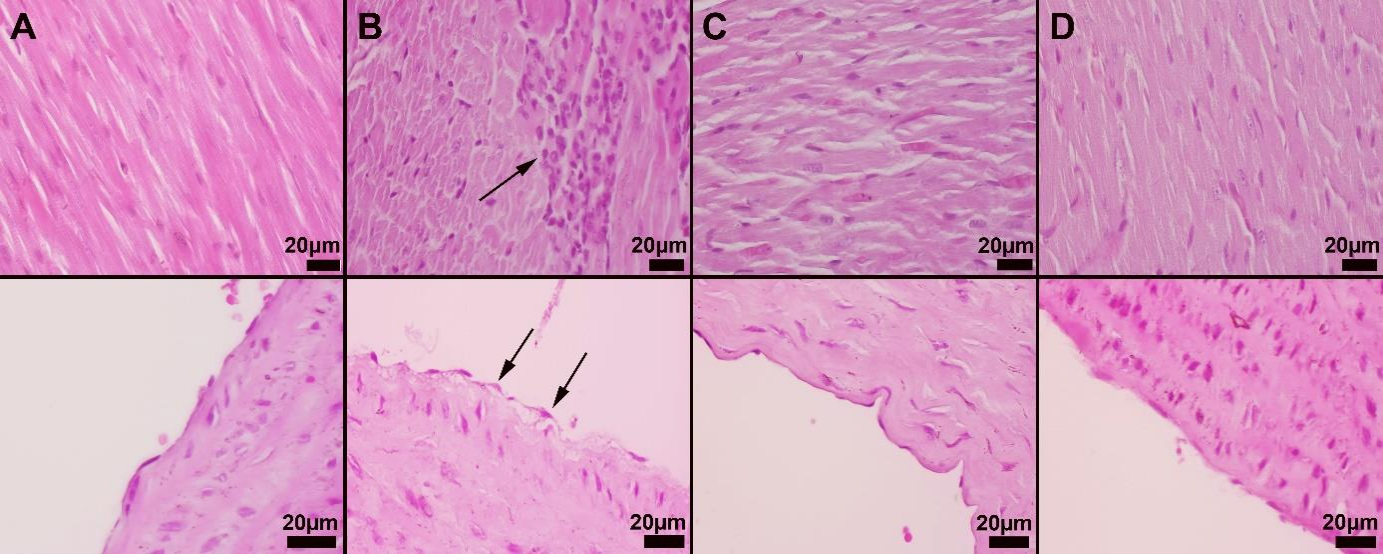
Result of histopathological changes

Histopathological examination of the cardiac and aortic tissues showed marked hyperemia and myocardial cell degenerations were noticed in the CP group. In addition, inflammatory cell infiltrations consisting mainly of lymphocytes were observed in rats in the CP group with varying severity. Marked amelioration was observed in the pathological findings in the CP+IRB group. Aortas in the CP group showed endothelial damage and desquamation (Figure 3). 

*Immunohistochemical results*

**Figure 4:**
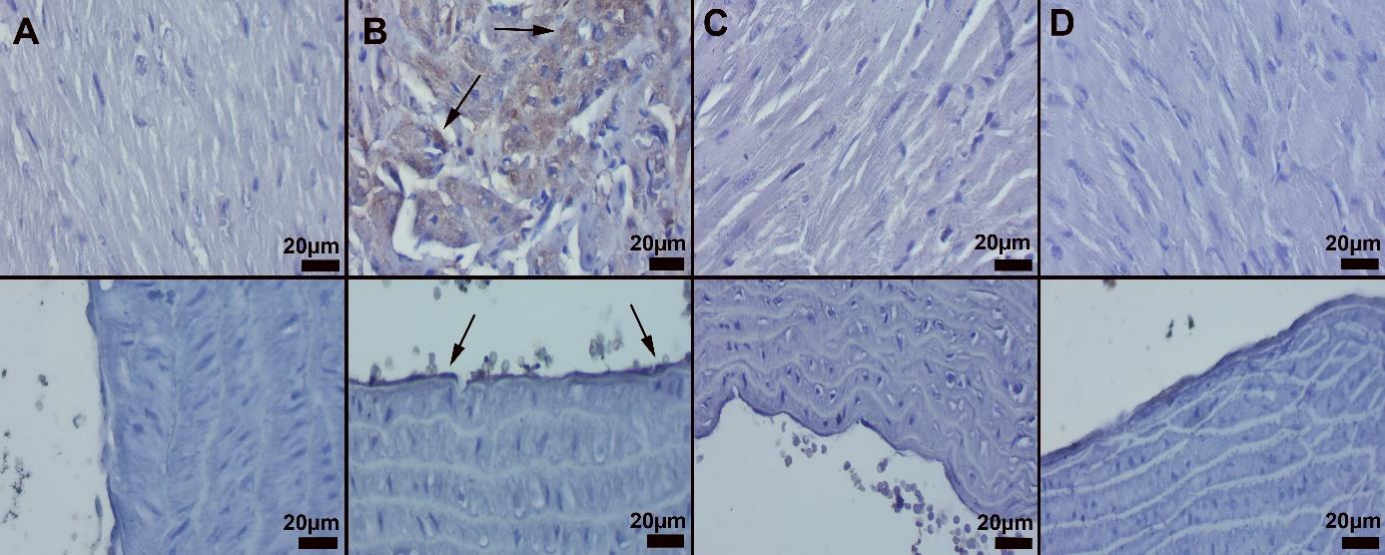
Result of Caspase-3 immunohistochemical activities

**Figure 5:**
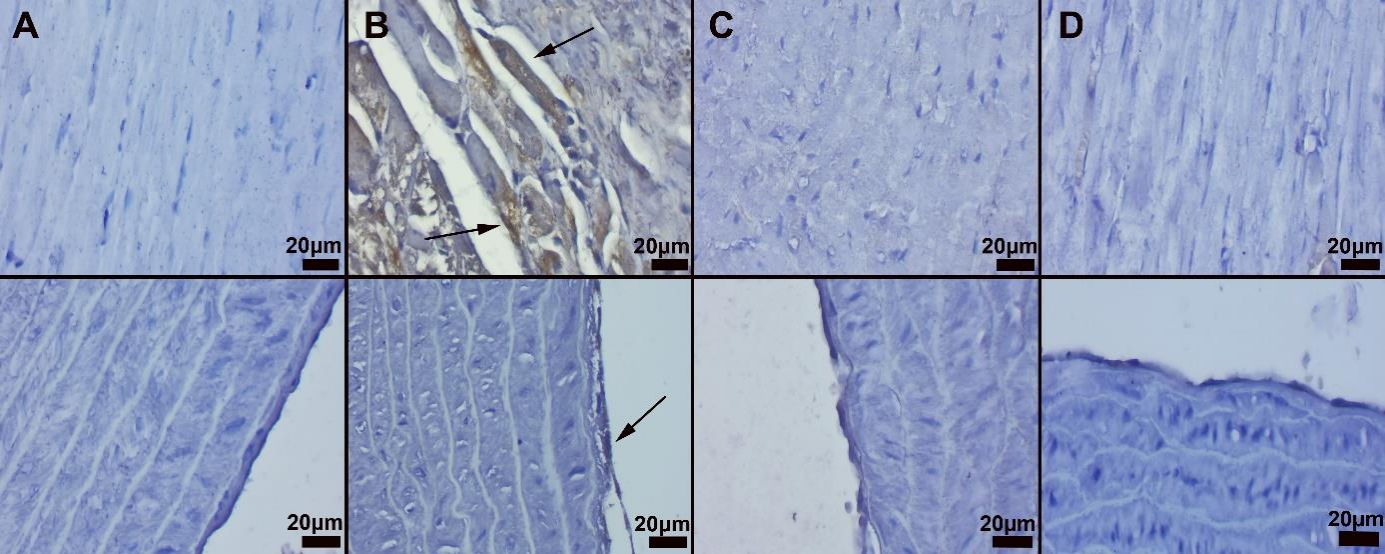
Result of VEGF immunohistochemical activities

Immunohistochemical results showed elevated Cas-3 and VEGF levels in endothelial and myocardial cells of the CPN group. IRB treatment caused amelioration in the CP+IRB group (Figures 4 and 5). Statistical analysis results are shown in Table 1. 

Values of CK-MB (A) and LDH (B). Comparison between groups was assessed by one-way ANOVA test followed by *post hoc* Tukey’s multiple comparisons test. Values are represented as means±SD. Creatine kinase MB (CKMB) and lactate dehydrogenase (LDH).***P<0.001, **P<0.01, * *P<*0.05, ‘*’ represents comparison with the control group, ###*P<*0.001, ## *P<*0.01, #P<0.05, ‘#’ represents comparison with CP group, ƟƟƟ *P<*0.001, ƟƟ*P<*0.01, Ɵ*P<*0.05, ‘Ɵ’ represents comparison with CP+IRB group

CP: cisplatin; IRB: Irbesartan

The total RNA was prepared and transcriptional levels of VCAM-1 (A) and NOX-1 (B) genes were evaluated by qRT-PCR. Y-axis represents the values of 2-∆∆CT. Each test was repeated four times. Comparison between groups was assessed by one-way ANOVA test followed by *post hoc* Tukey’s multiple comparisons test. Values are represented as means±SD.***P<0.001, **P<0.01, *P<0.05, ‘*’ represents comparison with control group, ###*P<*0.001, ##*P<*0.01, #*P<*0.05, ‘#’ represents comparison with CP group, ƟƟƟ*P<*0.001, ƟƟ*P<*0.01, Ɵ*P<*0.05, ‘Ɵ’ represents comparison with CP+IRB group

CP: cisplatin; IRB: Irbesartan; VCAM-1: Vascular cell adhesion molecule-1; NOX-1, NADPH oxidase-1

Histopathological appearance of the heart (upper row) and aorta (below row) among the groups. (A) Normal tissue histology in the control group. (B) Marked inflammatory cell infiltration (arrow) in the myocardium and endothelial cell desquamations in the aorta (arrows) in the CP group. (C) Decreased pathological findings in CP+IRB. (D) Normal tissue architectures in IRB groups, HE, scale bars=20 µm

Caspase-3 immunohistochemistry findings of hearts (upper row) and aortas (below row) between the groups. (A) Negative expression in the control group, rat No:2. (B) Marked increase in myocardial and endothelial cells (arrows) in CP group, rat No:1. (C) Decreased expression in CP+IRB group, rat No:4. (D) No expression in IRB group, rat No:2, Streptavidin biotin peroxidase method, scale bars=20 µm

**Table 1:** Immunohistochemical results of statistical analyses between the groups

	Control	CP	CP + IRB	IRB	*P*-value
Cas-3 heart	0.25±0.16^a^	2.00±0.75^b^	0.62±0.51^a^	0.12±0.12^a^	0.001
Cas-3 aorta	0.12±0.12^a^	1.87±0.83^b^	0.50±0.18^a^	0.25±0.16^a^	0.001
VEGF heart	0.25±0.16^a^	1.75±0.70^b^	0.50±0.18^c^	0.25±0.16^a^	0.001
VEGF aorta	0.12±0.12^a^	1.75±1.03^b^	0.50±0.18^a^	0.37±0.18^a^	0.001

*: Data expressed mean±standard deviation (SD). One-way ANOVA Duncan test

**: The differences between the groups carrying different letters in the same row are statistically significant, *P<*0.001

VEGF immunohistochemistry findings in the heart (upper row) and aorta (below row) among the groups. (A) No expression in the control group, rat No:5. (B) Marked increase in myocardial and endothelial cells (arrows) in CP group, rat No:1. (C) Decreased expression in CP+IRB group, rat No:3. (D) Negative expressions in IRB group, rat No:1. Streptavidin biotin peroxidase method, scale bars=20 µm

## Discussion

The present study demonstrated that IRB reduced VCAM-1, NOX-1, VEGF expression, and Cas-3 activation. These results showed that IRB had anti-inflammatory and antiapoptotic effects via reducing renin-angiotensin system (RAS) and MAPK activation. Together, IRB improved CP-induced cardiovascular toxicity and preserved cardiac function. 

CP, a chemotherapeutic agent, is extensively used to treat several solid organ cancers; however, unluckily, it causes cardiovascular toxicity that frequently needs dose modification or drug withdrawal (1-3, 26, 27). Additionally, the adverse effects of CP were associated with poor outcomes (3). However, cardiovascular toxicity such as hypertension and atherosclerosis of CP limit its use (1, 4, 14, 15, 28).

Several agents have been investigated in the literature to prevent cardiotoxicity related to CP. In these studies, antioxidant, anti-inflammatory, and antiapoptotic mechanisms on CP-induced damage mechanisms were investigated (13, 14, 29). Research showed that lactobacillus supplementation improved cardiac function in cardiotoxicity related to CP by its anti-inflammatory effect (29). A study demonstrated that superoxide dismutase-2 (SOD-2) increased cardiotoxicity related to the CP group, and acetyl-l-carnitine decreased the levels of SOD-2 (13). Gunturk *et al*. reported that N-acetylcysteine improved cardiac function and histological damage in CP-induced cardiotoxicity (14). The antioxidant effects of acetyl-l-carnitine and N-acetylcysteine were emphasized in the last two articles (13, 14).

After myocyte damage, creatine kinase (CK), CK-MB, and LDH levels increase. In the current study, the increased CK-MB and LDH levels in rats treated with CP were significantly decreased in rats treated with IRB+CP. Additionally, our histological findings were consistent with our biochemical results. AngiotensinII (Ang II) is the final product of RAS and has an essential role in this system by contracting blood vessels. Researchers (30) showed that CP-based chemotherapy activated RAS. Increased RAS activity may explain the vascular and cardiac destructive effects of CP-based chemotherapy. However, Angiotensin II has been shown to worsen the inflammatory state by increasing the transcription factor nuclear factor kappa-B (NFKβ) activity, proinflammatory molecules expression such as VCAM-1, and the release of cytokines such as interleukin-6 (31, 32). VCAM-1, an endothelial cell surface glycoprotein whose expression is activated by proinflammatory cytokines, is involved in the extravasation of leukocytes in inflammation. Therefore, the levels of VCAM-1 increase in inflammatory diseases (11, 12).

Furthermore, Candido *et al*. reported that VCAM-1 levels increased in the aortas of diabetic apoE-null mice, and these increases were ameliorated with IRB(33). The current study showed that VCAM-1 level increased in rats exposed to CP, while VCAM-1 level decreased statistically significantly in rats given CP plus IRB. The increase in VCAM-1, which is involved in the adhesion of leukocytes to the cell, has shown increased inflammation in rats exposed to CP. The decrease in VCAM-1 level in rats treated with IRB plus CP demonstrated that IRB prevented the extravasation of leukocytes.

Ang II works together with the NOX enzyme system in the vasculature. Localization, differential tissue distribution, and subcellular compartmentalization probably have an essential role in NOX-specific actions triggered by Ang II (34). NOX-1 is involved in cell growth, cell signaling, motility, blood pressure regulation, and angiogenesis (35-37). Although NOX-1, primarily expressed on endothelial cells of the vascular wall, has been associated with several vascular diseases, including hypertension, restenosis, atherosclerosis, and vascular remodeling, its role in cardiac disease is not fully elucidated because of the relatively low expression of NOX-1 in cardiac tissues.

On the other hand, a study showed that NOX-1 was involved in endotoxin-induced apoptosis(38). Furthermore, Jiang *et al.* demonstrated that NOX-1 was necessary for tumor necrosis factor-α and NFK-β associated myocardial injury in late myocardial ischemic preconditioning (39). Previous studies showed that NOX-1 up-regulation was responsible for CP-induced kidney injury (39), and NOX-1 inhibition protected renal ischemia-reperfusion ınjury through oxygen-derived ROS suppression and related mechanisms (especially ROS-mediated ERK signaling)(40). The present study demonstrated that NOX-1 levels increased in rats exposed to CP, while NOX-1 levels decreased statistically significantly in rats given CP plus IRB. Ang II induces NOX-1 activity in smooth muscle cells (41). IRB may have benefited by suppressing increased Ang II levels and decreasing NOX-1 levels in smooth muscle cells. This study is the first, to the best of our knowledge, that elucidated the association between CP-related cardiovascular toxicity and NOX-1 up-regulation.

Caspases are cysteine-protease group enzymes that play an essential role during apoptosis. The increase in Cas-3 in parallel with the increase in VEGF levels, which is one of the critical mediators of inflammation in both heart tissue and aortic tissue, has also proven that inflammation is accompanied by apoptosis (42-44). The anti-inflammatory and antiapoptotic effects of IRB against the progressive damage of the tissue by inflammation and apoptosis, reducing inflammation and apoptosis, and improving the damage indicate that the drug has anti-inflammatory and antiapoptotic properties besides its antioxidant effect (21, 45).

In the literature, although articles investigated the renal, vascular, etc., protective effects of IRB, there are various studies associated with the protective effects of IRB on cardiovascular toxicity of CP that are limited (21, 45-48). Researchers showed that IRB with antioxidant, anti-inflammatory, and antiapoptotic effects prevented cardiotoxicity developed with doxorubicin (21). Boccellino *et al*. demonstrated IRB’s protective effect on myocardial damage via reducing oxidative stress and inflammation in myocardial damage caused by hypoxia. A study showed the antioxidative and antiapoptotic protective efficacy of IRB in myocardial damage in diabetic rats (49).

Endothelial cell desquamation refers to the detachment and shedding of endothelial cells from the vascular wall, which can occur during vascular injury and may be an indicator of a pathological condition such as diabetes (50). Endothelial cell desquamation was observed in the group treated with CP. This finding suggests that cis treatment may cause vascular injury. IBR treatment significantly improved this pathological finding.

## Conclusion

As a result of the current study, inflammation, increase in angiotensin II activity, increase in the NOX enzyme system activity, and apoptosis play an essential role in CP-induced cardiovascular toxicity. Furthermore, increased VEGF levels detected immunohistochemically showed increased inflammation, and increased caspase levels showed apoptosis. As a result, it has been demonstrated in this study that IRB, which is used as an antihypertensive due to its angiotensin receptor blocker feature, can prevent cardiotoxicity by inhibiting the harmful effects of Ang II, preventing leukocyte migration, reducing oxidative stress, inflammation, and apoptosis.

IRB can be considered a potential therapeutic agent in both preventing and treating undesirable cardiovascular complications such as hypertension that may develop due to CP. However, detailed studies on this subject are needed.

## Authors’ Contributions

DU K designed the experiments; DU K, A M, HI B, and E A performed experiments and collected data; DUK, S O, and F A discussed the results and strategy; DU K and S O supervised, directed, and managed the study; DU K, S O, E A, HI B, A M, and F A approved the final version to be published. 

## Conflicts of Interest

The authors have no conflicts of interest to declare.
